# Ectopic adrenocortical adenoma characterized by hypogonadism: a case report and review of the literature

**DOI:** 10.1186/s13256-024-04595-z

**Published:** 2024-06-12

**Authors:** Zhihua Wang, Xueyu Zhong, Jiayu Yu, Huiqing Li, Juan Zheng

**Affiliations:** Department of Endocrinology, Union Hospital, Tongji Medical College, Huazhong University of Science and Technology, Wuhan, 430022 China

**Keywords:** Ectopic adrenocortical adenoma, Cushing’s syndrome, Hypogonadotropic hypogonadism, Functional localization, Case report

## Abstract

**Background:**

Currently, there is a scarcity of cases and diagnostic data regarding ectopic adrenocortical adenomas, particularly in relation to their impact on gonadal function and localization diagnostic techniques. We report a typical case of ectopic adrenocortical adenomas and the data of treatment follow-up, and review the literature of 31 available cases of ectopic adrenocortical adenomas.

**Case presentation:**

A 27-year-old Chinese female patient was admitted to our hospital for hypertension, hyperglycaemia and primary amenorrhea. The patient was functionally diagnosed with ACTH-independent CS and hypogonadotropic hypogonadism. Radiological evaluations, including Computed Tomography (CT) and functional imaging, identified a mass at the left renal hilum. Histological assessments post-surgical excision confirmed the mass to be an ectopic adrenocortical adenoma. A subsequent 3-month follow-up showed no signs of disease recurrence, a swift recovery of the cortisol axis was observed, with a partial recuperation of the gonadal axis. Review: Our literature review shows that the most common ectopic areas of cortisol adenomas are renal hilum and hepatic region. The most positive biomarker is Melan A, and only a few cases have been diagnosed with functional localization.

**Conclusion:**

Ectopic adrenocortical adenomas may be asymptomatic in the early stage and can impact gonadal function. Physicians who treat hypogonadism must be aware of the need to test cortisol levels and perform functional localization in patients with lumps present.

## Background

Cushing’s syndrome (CS) is a chronic pathological hypercortisolism, which can be either endogenous or exogenous [1]. Endogenous CS can be divided into adrenocorticotropic hormone (ACTH)-dependent (pathological increase in ACTH secretion promotes cortisol production) or ACTH-independent (excessive autonomous secretion of cortisol by adrenal tissue) [2]. ACTH-independent CS accounts for about 20% of endogenous causes, more commonly caused by adrenal adenoma, adrenal cortical carcinoma, and in rare cases, by nodular adrenal hyperplasia or primary pigmented nodular adrenal disease [3]. The occurrence of functional ectopic adrenal cortical adenoma, is a seldom-seen origin of autonomous CS and defined as isolated benign adrenal cortical tissue outside orthotopic adrenal glands. These adenomas predominantly localize in areas such as the renal hilum, vertebral body, and liver, with a minority found in the gastric antrum and ovaries. Such presentations often culminate in classical manifestations of CS. Upon diagnosing CS via pertinent hormone assays and other diagnostic measures, it becomes imperative to ascertain its etiology. Especially, discerning the functionality of adrenal tumor holds pivotal importance in directing toward a definitive surgical intervention.

While it’s recognized that elevated cortisol levels can have repercussions on gonadal function, the specific influence of hypercortisolemia on gonadal glands in patients with ectopic adrenocortical adenoma remains ambiguous, given the rarity of such cases. Drawing from investigations centered around pediatric and adolescent CS, we aim to elucidate its impact on the gonadal axis. This report encompasses a review of ectopic adrenocortical adenoma cases, integrating insights from our clinical encounters with functional ectopic adrenocortical adenoma with hypogonadotropic hypogonadism.

## Case presentation

A 27-year-old Chinese female patient from a southern Chinese town presented with primary amenorrhea, having not menstruated before the age of 18 in 2014. She was chromosomally identified as 46, XX, and ultrasonography indicated an infantile uterus. She was diagnosed with primary amenorrhea and showed menstrual onset when treated artificial menstrual cycle therapy with estradiol 1 mg and dydrogesterone 10 mg. Over the past 6 months, she experienced hair loss, fatigue, polydipsia, nocturia, palpitations, thin and reddened skin, muscle weakness, difficulty standing up from a squatting position, darkened periorbital skin pigmentation, and increased blood pressure. Antihypertensive medications did not effectively control her blood pressure. She had no history of smoking or alcohol consumption. Physical examination revealed: height 160 cm, weight 68 kg, BMI 23.44, blood pressure 147/103 mmHg, pulse 128 bpm, and temperature 36 °C. She exhibited a moon face, a plethora of appearance, coffee-colored periorbital pigmentation, thin cheek skin with visible superficial blood vessels, buffalo hump, central obesity, and ecchymosis on the shin. Neurological examination was unremarkable.

The summary of the patient's laboratory tests is shown in Tables 1 and 2. Both the low-dose dexamethasone suppression test (DST) and the high-dose DST failed to suppress cortisol production. The decreased gonadotropins and sex hormones indicated ACTH-independent CS accompanied by hypogonadotropic hypogonadism.

**Table 1:** Changes in hormone levels

Variables	Reference range	Age 18	At admission	After surgery	3-month follow-up
ACTH (pg/ml) (8AM)	7.00–64.00	NA	2.86	4.79	76.38
Cortisol (ug/L) (8AM)	37.0–194.0	NA	191.0	< 0.8	33.0
24 h UFC (ug/24 h)	4.3–176.0	NA	360.0	NA	< 1.0
LH (IU/L)	1.80–11.78	1.64	0.97	NA	2.55
FSH (IU/L)	3.00–8.10	2.26	2.98	NA	4.60
E2 (pg/mL)	21.0–251.0	7.5	< 10.0	NA	< 10.0
P (ng/mL)	< 0.30	0.61	0.20	NA	< 0.10
T (ng/mL)	0.11–0.57	31.07	0.57	NA	0.08

At the time of admission, artificial menstrual cycle therapy had been discontinued for 4 months; After the operation, oral prednisone 12.5 mg was gradually reduced. Three months post-surgery and the withdrawal of glucocorticoid therapy; LH: luteinizing hormone; FSH: Follicle-stimulating Hormone; UFC: urinary-free cortisol; NA: not available

**Table 2:** Laboratory values

Test	Result	Reference range
WBC (10^9^/L)	7.56	3.5–9.5
NE (10^9^/L)	5.34	1.8–6.3
HGb (g/L)	135	115–150
HCT (%)	41.7	35–45
PLT (10^9^/L)	258	125–350
AST (U/L)	30	8–40
ALT (U/L)	33	5–35
Total bilirubin (μmol/L)	7.5	5.1–19.0
Urea (mmol/L)	4.20	2.9–8.2
Cr (μmol/L)	58.7	44.0–106.0
INR	0.89	0.80–1.20

*WBC* White Blood Cells, *NE* Neutrophils, *HGb* Hemoglobin, *HCT* Hematocrit, *PLT* Platelets, *AST* Aspartate aminotransferase, *ALT* Alanine aminotransferase, *Cr* Creatinine, *INR* International Normalized

**Fig. 1:**
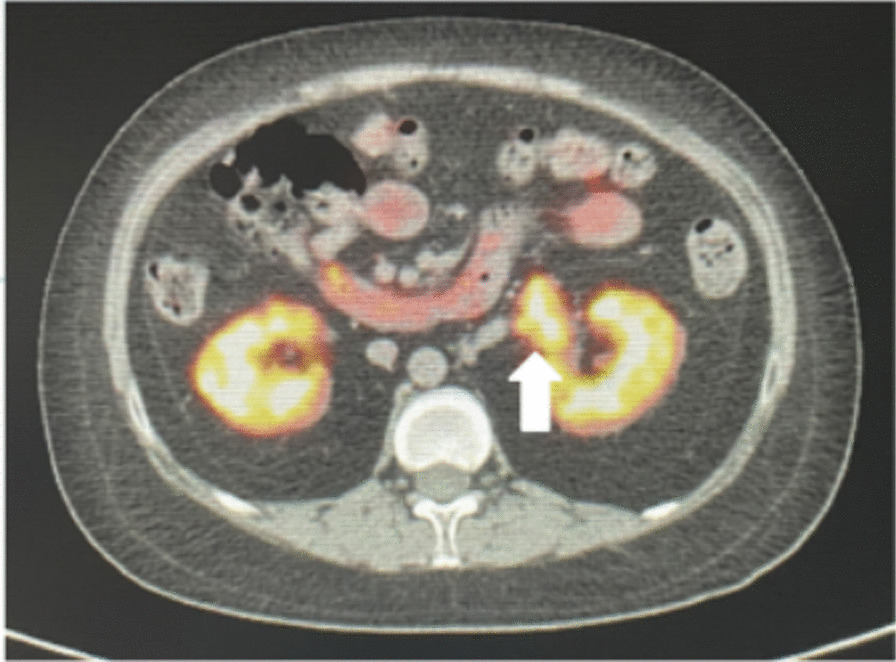
^68^Ga-DOTA-TATE-PET/CT images show a mass with significant enhancement in the left renal hilum (arrows)

Gynecological ultrasound examination results are summarized in Table 3. No obvious abnormalities were observed in the pituitary MRI. Contrast-enhanced abdominal computed tomography (CT) showed slender bilateral adrenal glands without any nodules or hyperplasia. A soft tissue density shadow measuring approximately 2.9 × 1.8 cm was observed in the left renal hilum. The somatostatin receptor PET/CT whole-body imaging (^68^Ga-DOTA-TATE imaging) showed abnormal tracer accumulation in the left renal hilum soft tissue mass (Fig. 1) indicating a somatostatin receptor-positive lesion. This led to the suspicion of an ectopic adrenal cortical adenoma at the left renal hilum.

**Table 3:** Gynecological B-mode ultrasound

Date(Year-month)	Uterine size (*L* × *W* × *T* cm)	Ovarian size (*L* × *W* cm)
2014–6	1.6 × 1.0 × 1.3	Poor display
2015–4	3.0 × 1.6 × 2.3	Left 1.5 × 0.8 Right 1.8 × 1.2
2017–9	3.1 × 1.8 × 2.7	Left 1.5 × 0.6 Right 1.3 × 0.7
2023–6	2.6 × 2.2 × 1.9	NA

The patient was given artificial menstrual cycle therapy from 2014–6

*NA* not available

During hospitalization, the patient was administered 30 mg of nifedipine sustained-release tablets, 5 mg of linagliptin, 10 mg of dapagliflozin, and 0.25 μg of calcitriol daily. Upon completion of the required examinations, she underwent laparoscopic tumor resection. The tumor was 2.5 × 2.0 × 1.0 cm in size and yellow–brown in color. Histopathological examination confirmed it to be an ectopic adrenal cortical adenoma. Immunohistochemical staining showed: a-Inhibin(+), Calretinin(+), MelanA(+), Syn(+), CgA(−), CD99(−), s-100(−), EMA(−), CK8/18(−), P53(weakly positive in some areas), and Ki67(LI: about 1%). One week after surgery, the morning plasma cortisol level dropped to < 0.8 ug/L and the plasma ACTH level rose to 4.79 pg/ml. After the operation, the patient was given oral prednisone 12.5 mg everyday, which was gradually reduced. No obvious adverse reactions occurred after strict adherence to the medication regimen and regular review. Three months post-surgery and the withdrawal of glucocorticoid therapy, the level of blood cortisol was 33.0 ug/L, ACTH was 76.38 pg/ml. ^68^Ga-Pentixafor PET/CT imaging (CXCR4 receptor imaging) did not reveal any significant abnormal concentrations. In our case, three months after the surgery to remove the ectopic cortisol adenoma and after the hypercortisolemia state, a follow-up revealed some recovery of the gonadal axis. The level of LH and FSH increased, and the GnRH excitation test could be excited. The ACTH excitation test was not fully excited, suggesting that the function of the adrenal cortex needs to be further restored. The patient had urgent fertility needs and relatively deficient ovaries. We subsequently gave GnRH pulse therapy, and the gonads gradually recovered and menstruation gradually appeared regularly.

## Literature review

To date, 31 cases of ectopic adrenal cortical adenomas have been reported (Table 4), of which 10 were ectopic adrenal cortical adenomas outside the renal hilum (33.3%), followed by the vertebral region (30%) and liver area (33.3%). Individual cases were found in the gastric antrum and ovaries. The patient's age ranged from 5 months to 75 years, with the largest diameter ranging from 8.5 to 8.8 cm. Among them, 19 were non-functional ectopic adrenal cortical adenomas. Most of cases are pathological diagnosis after operation, but lack of endocrine function assessment. In the cases with detailed pathological results, the positive rates of biomarkers expressed in adrenal cortical cells were: Melan A (79%), A-inhibin (63%), CYP17 (11%), CYP11B1 (5%), SF-1 (5%); The positive rates of these markers were Syn (68%), CD56 (16%), HSD3B2 (5%).

**Table 4:** The case reports of ectopic adrenocortical tumor

Nr	Age (y)	size(cm)	Location	Cortisol level detection	HDDST	Adrenal gland	preoperative localization	Pathological findings(+)
1 [10]	57	3.1 × 2.7	Left renal hilum	Elevated	Uninhibition	Atrophy	CT, AVS, 18F-FDG PET/CT	Pathological diagnosis
2 [5]	27	2.7 × 2.7	Left renal hilum	Elevated	NA	Atrophy	CT	Melan A, a-Inhibin, Vimentin, NSE, Ki67 < 1%
3 [29]	60	2.3 × 2.3	Right renal hilum	NA	NA	Normal	CT, MRI	Pathological diagnosis
4 [7]	63	3.5 × 3.5	Left renal hilum	Elevated	Uninhibition	Normal	CT, MRI	Lots of smooth endoplasmic reticulum and mitochondria with tubular cristae
5 [8]	38	3.5 × 5.3	Left renal hilum	Elevated	Uninhibition	Atrophy	Not described	Pathological diagnosis
6 [9]	53	3.5 × 3.0	Left renal hilum	Elevated	Uninhibition	Atrophy	CT	Melan A, CYP17, HSD3B2
7 [30]	37	2.9 × 2.8	Right renal hilum	Elevated	NA	Normal	CT	Syn, CD56, Vimentin, Ki67 < 2%
8 [31]	33	3.5 × 3.5	Left renal hilum	NA	NA	Normal	CT, MRI	CYP11B1, CYP17
9 [8]	47	1.7 × 1.5	Left renal hilum	NA	NA	Normal	CT, MRI	Vimentin, Syn, CD56, Chromogranin
10 [32]	57	2.3 × 2.2	Left renal hilum	NA	NA	Normal	CT, MRI	SF-1, Melan A, Syn, Ki67 < 3%
11 [33]	72	2.0 × 3.0	The lesser curvature of the stomach	NA	NA	Normal	CT, Fibergastros-copy	Melan A, CD34,
12 [34]	8	1.9 × 2.4	L2	NA	NA	Normal	MRI	Pathological diagnosis
13 [35]	16	2.5 × 1.9	L2 Cauda equina	NA	NA	Normal	X-ray	Lots of smooth endoplasmic reticulum and mitochondria with tubular cristae
14 [36]	27	3.0 × 2.0	Cauda equina	NA	NA	Normal	CT	a-Inhibin, Melan A, Syn
15 [37]	27	3.6 × 2.3	Medullary cone	NA	NA	Normal	MRI	a-Inhibin, Melan A, Syn
16 [38]	44	2.5 × 2.5	L1	NA	NA	Normal	MRI	Melan A, a-Inhibin, Syn, Ki67 < 3%
17 [39]	5 mo	6.0 × 1.5	T10–L2	NA	NA	Normal	MRI	a-Inhibin, Melan A, Syn
18 [40]	1	5.5 × 2.0	T12–L1, L3–L4	NA	NA	Normal	CT, MRI	a-Inhibin, Syn, Vimentin,
19 [41]	8	6.0 × 2.8	L 3–L5	Normal	Uninhibition	Normal	NA	Melan A, Syn, CD56, Ki67 < 3%,
20 [42]	46	3.0 × 2.5	T12–L1	NA	NA	Normal	MRI	a-Inhibin, Melan A, Syn, Vimentin, Ki67 < 1.5%
21 [13]	40	5.7 × 4.2	Right ovary	Elevated	Uninhibition	Atrophy	CT, AVS, SRS	Pathological diagnosis
22 [6]	55	2.5 × 2.5	Right lobe of the liver	NA	NA	Normal	CT, MRI	Pathological diagnosis
23 [43]	66	1.5 × 1.5	Right lobe of the liver	NA	NA	Normal	CT	Pathological diagnosis
24 [44]	62	3.0 × 3.0	Right lobe of the liver	NA	NA	Normal	CT	Pathological diagnosis
25 [45]	45	2.5 × 2.5	Right lobe of the liver	NA	NA	Normal	CT, MRI	Pathological diagnosis
26 [46]	56	3.4 × 3.4	Right lobe of the liver	NA	NA	Normal	CT, MRI	a-Inhibin, Melan A, Syn,
27 [46]	75	4.5 × 2.2	Right lobe of the liver	NA	NA	Normal	CT	a-Inhibin, Melan A
28 [46]	64	2.6 × 2.6	Right lobe of the liver	NA	NA	Normal	CT	Melan A, a-Inhibin, Syn
29 [47]	75	2.5 × 2.5	Right lobe of the liver	NA	NA	Normal	CT	Melan A, a-Inhibin
30 [48]	44	8.8 × 8.5	Right lobe of the liver	NA	NA	Normal	CT	a-Inhibin, Melan A, Syn, Ki67 < 3%,
31 [47]	59	1.5 × 1.5	Right lobe of the liver	NA	NA	Normal	18F-FDG-PET/CT, CT, MRI	Pathological diagnosis

*HDDST* high-dose dexamethasone suppression test, *AVS* Adrenal venous blood sample, *SRS* Somatostatin receptor scintigraphy, *CT* Radiological evaluations using Computed Tomography, *Pathological findings(+)* Pathological immunohistochemical tests were positive biological markers, *Pathological diagnosis* Biomarkers for immunohistochemical testing were not reported in detail in this case, *NA* not available

## Discussion

Ectopic adrenocortical adenomas is an extremely rare cause of ACTH-independent CS. The localization diagnosis, especially the functional localization diagnosis, is very important for the subsequent treatment of the disease but is often overlooked due to the disease’s insidious onset. Among the 31 reported cases, only a few performed functional localization diagnostics. Our case involves a 27-year-old Chinese female with ACTH-independent CS and primary amenorrhea due to gonadotropin deficiency. This is the first case to use ^68^Ga-DOTA-TATE for the functional localization diagnosis of an ectopic adrenal cortical adenoma. Post-tumour resection follow-up using ^68^Ga-pentixafor PET/CT confirmed complete removal of the cortisol-producing tumour, with subsequent recovery of adrenal and gonadal function. Thus, early diagnosis must include functional localization tests, which effectively identify high-endocrine adrenal lesions, enabling early diagnosis and treatment to prevent abnormal gonadal axis development due to delayed treatment.

### Localization diagnosis of ectopic cortisol adenoma

During embryonic growth, ectopic adrenal tissue can exist along the path from the gonad to the adrenal gland. It is reported that 50% of newborns and 1% of adults have ectopic adrenal tissue [4], and the clinical characteristics of ectopic adrenal tissue are determined by its hormonal secretion status. Most ectopic adrenal tissues will atrophy; a small part may proliferate, leading to increased cortisol secretion [5]. Ectopic adrenal cortical adenoma is an extremely rare cause of ACTH-independent CS. Common anatomical locations of published ectopic adrenal tissue include the cisterna chyli, kidneys, broad ligament, epididymis, and testicles. The brain, lungs, and stomach are also rare sites for ectopic adrenal tissue [6]. Besides, in most children, the onset of CS is insidious. Metabolic conditions like hyperglycemia and hypertension may go unnoticed. Hypertension is found in 58–85% of adults [7] and rather less frequently (47–49%) in children [8, 9] with Cushing’s syndrome. Therefore, the patient in this case was not diagnosed at an early stage.

The localization diagnosis of CS holds paramount importance for subsequent treatment and the prevention of disease progression. Screening and diagnostic tests for CS assess cortisol secretory status: abnormal circadian rhythm with late-night salivary cortisol (LNSC), impaired glucocorticoid feedback with overnight 1 mg DST or low-dose 2-day dexamethasone test (LDDT), and increased bioavailable cortisol with 24-h urinary free cortisol (UFC). In this setting, the sensitivity of all tests is higher than 90%; the highest sensitivity rates are obtained with DST and LNSC and the lowest with UFC. Specificity is somewhat lower than sensitivity, with LNSC being the most specific and DST and UFC being the least specific [10].

CT scanning is a sensitive method for localizing ectopic adrenal tumors. More than 70% of adrenal cortical adenomas contain a high intracellular lipid content. When CT values are less than 10HU, they can be determined as lipid-rich adrenal adenomas, eliminating the need for further imaging studies. If the intracellular lipid content of the adenoma is relatively low and cannot be determined by CT, MRI can better detect lesions within the fat. Studies indicate that when unenhanced CT reveals adrenal lesions with CT values between 10-20HU, 62% of the lesions can be determined as lipid-rich adrenal adenomas through quantitative chemical shift MRI [11, 12].

For the treatment of functional ectopic adrenal tissue, radical excision surgery is the recommended approach. However, confirming the hormonal secretion status of the tumor pre-surgery poses a challenge. Selective venous sampling is a highly sensitive method for localizing various neuroendocrine tumors, distinguishing the functional status of adrenal tumors, and determining the functional dominant side. Adrenal venous blood sampling (AVS) can be used to localize high-functional nodules in patients with ACTH-independent CS. There have been two reported cases from Peking Union Medical College Hospital using venous blood sampling to locate ectopic adrenal cortical adenomas: one was a 57-year-old female with a left renal hilum tumor associated with CS [10], and the other was a 40-year-old female with a right ovarian tumor associated with CS [13]. Both were successfully guided to undergo surgical treatment. Its most crucial and widespread use is to localize primary aldosteronism (PA) [14], with detection sensitivities and specificities of 95% and 100%, respectively [14, 15]. However, adrenal venous blood sampling has technical challenges. Success rates vary significantly between institutions, ranging from 42 to 96% [16], and depend on the physician’s skills and experience, with inconsistent operational methods and result assessments.

^68^Ga-DOTA-TATE is a selective somatostatin receptor PET tracer used to assess the functional imaging of well-differentiated neuroendocrine tumors (NETs) which is solid tumor that originate from peptide-capable neurons and neuroendocrine cells. It has become the preferred imaging modality for initial diagnosis and localization of unknown primary tumors [17]. Currently, this technique is mainly used for ectopic ACTH-dependent CS. One study showed that of 27 children and adolescents with ACTH-dependent CS who had ^68^Ga-DOTA-TATE-PET/CT done, 18 (66%) had positive results [18]. However, there have been no reports of its use for the localization diagnosis of ectopic adrenal cortical adenomas. In our case, a preoperative ^68^Ga-DOTA-TATE-PET/CT showed a positive somatostatin receptor lesion in the left renal hilum area. Histopathological examination confirmed this lesion to be an ectopic adrenal cortical adenoma. It suggests that ^68^Ga-DOTA-TATE-PET/CT can be used to identify ectopic adrenal cortical adenoma.

Ectopic adrenal cortical adenoma may be classified as NETs. Immunohistochemical staining serves as a vital tool for distinguishing the nature of such tumors. SF-1 is deemed the most reliable and specific biomarker indicative of adrenal origin, and another widely used biomarker with high specificity and sensitivity is Melan A [19]. According to the 2022 WHO Classification of Endocrine and Neuroendocrine Tumors, the vast majority of NETs express biomarkers such as INSM1, CgA, and Syn [20]. In our analysis of 31 cases of ectopic adrenal cortical adenomas, the presence of adrenal origin biomarker SF-1 (0/0), Melan A (15/15) and for biomarkers indicative of neuroendocrine tumor origin CgA (0/12), Syn (13/13), INSM1 (0/0)-with parenthetical notation indicating that (number of cases positive for the biomarker/total number of cases tested for the biomarker). Among all cases, Melan A demonstrated a 100% positive rate for adrenal origin, whereas CgA showed a 0% positive rate, and INSM1 was not tested. Although Syn showed a 100% positive rate in our cases, it is also frequently detected in adrenal cortical tissues, suggesting its lower specificity for NETs [19]. Therefore, despite potential indications of NETs, these cases cannot be definitively classified as NETs based on current definitions. Immunohistochemical staining of ectopic adrenal cortical adenoma in our case showed: a-Inhibin(+), Calretinin(+), MelanA(+), Syn(+), CgA(−), CD99(−), s-100(−), EMA(−), CK8/18(−), P53(weakly positive in some areas), and Ki67(LI: about 1%). These findings also do not suffice for a NETs classification.

Beyond ^68^Ga-DOTA-TATE-PET/CT, recent reports indicate that the CXCR4-specific PET tracer ^68^Ga-pentixafor demonstrates tremendous potential in identifying functional adrenal cortical adenomas. ^68^Ga-pentixafor PET/CT, with an SUVmax > 8.6, offers a 100% accuracy rate in distinguishing patients' functional cortisol adenomas from adrenal hyperplasia and non-functional adrenal adenomas [21]. This method effectively identifies high-endocrine adrenal lesions, especially smaller lesions that are difficult to detect and distinguish on conventional CT imaging, guiding precise lesion resection. For multiple adrenal lesions, it can determine the dominant side with endocrine function, guiding the surgical decision of patients with multiple adrenal lesions. After tumor resection, it can also serve as a method to judge the efficacy of surgical treatment. In our case, since our unit did not have the conditions to perform ^68^Ga-pentixafor PET/CT at the time of diagnosis, this examination was not performed before surgery. Three months after the operation, a follow-up ^68^Ga-pentixafor PET/CT was performed, and no lesions with high focal uptake were found. At this time, the patient did not have a recurrence of CS, confirming that the cortisol tumor in the patient’s body had been completely removed. In addition, ^68^Ga-pentixafor PET/CT can be used to inspect aldosterone-producing tissues. Some studies have shown that the sensitivity of ^68^Ga-pentixafor PET/CT in detecting aldosterone adenomas can reach between 88 and 100%, with a specificity ranging from 78.6 to 92% [21].

### The impact of hypercortisolism on the gonads

In our case, the patient had persistent symptoms of hypogonadotropic hypogonadism, primary amenorrhea, and ovarian dysplasia before surgery. After surgical removal of the cortisoloma to relieve the hypercortisolemia state, the GnRH excitation test could be excited, indicating partial but low recovery of gonadism, considering that the low LH and FSH levels in the patient in the past may be related to the inhibition of GnRH secretion from the hypothalamus by high cortisol levels since childhood. After all, in most children, the onset of CS is insidious. Metabolic conditions like hyperglycemia and hypertension may go unnoticed in the absence of overt CS symptoms and signs. Reports have shown that the average duration of symptoms before the diagnosis of pediatric CS ranges from 1.7 to 2.5 years [22]. Excess cortisol can impact gonadal hormones through various mechanisms, such as reducing the release of hypothalamic GnRH and pituitary LH. It can also decrease the gonadal response to LH and the concentration of LH receptors. In rhesus monkeys, supraphysiological doses of prednisolone suppress the secretion of gonadotropins, but this suppression is almost entirely reversed by intermittent GnRH infusion, indicating that cortisol can suppress GnRH secretion [23]. Under certain conditions, high levels of glucocorticoids produced via the HPA axis result in a reduction in the synthesis and release of gonadal hormones, inhibiting follicular development and ovulation in female animals [24]. Studies have shown that when glucocorticoid levels exceed normal levels, they act through glucocorticoid receptors to downregulate LH receptor expression in the ovaries [24]. Both women with Cushing's disease and those undergoing long-term supraphysiological glucocorticoid treatment have a reduced LH response to GnRH, suggesting that glucocorticoids also suppress pituitary gonadotropin's response to hypothalamic input [23].

Ectopic CS in children accounts for less than 1% of CS and is 80 times less frequent than Cushing’s disease [25]. Continuous hypercortisolemia will gradually affect the function and development of the gonads and related organs. In our case, the most evident symptoms were amenorrhea and underdeveloped uterus. Amenorrhea is one of the primary symptoms of pediatric CS. A retrospective study analyzed 59 children and adolescents with endogenous CS of various causes; 78% presented with primary/secondary amenorrhea, 38% with precocious puberty, and 3% with delayed puberty [26]. Elevated cortisol levels suppress estrogen's effects on the uterus. Studies have shown that hypercortisolism significantly inhibits uterine growth stimulated by estrogen. Firstly, cortisol might suppress uterine growth by inhibiting estrogen’s direct pro-growth effects on the genome. Secondly, cortisol might suppress the secretion of estrogen-dependent growth factors or induce uterine cell resistance to these growth factors. It might also be achieved by reducing the concentration of estrogen receptors [27]. In one series of 45 women with Cushing's disease, 33% presented with amenorrhea, which was associated with higher serum cortisol levels and lower estradiol and sex hormone-binding globulin levels [28]. Although some cases in this study had elevated androgen levels, amenorrhea was not related to serum androgen levels. Therefore, amenorrhea in Cushing's disease is more likely mediated by cortisol-suppressing GnRH than by hyperandrogenism.

In our case, three months after the surgery to remove the ectopic cortisol adenoma and after the hypercortisolemia state, a follow-up revealed some recovery of the gonadal axis. The patient had urgent fertility needs and relatively deficient ovaries. We subsequently gave GnRH pulse therapy, and the gonads gradually recovered and menstruation gradually appeared regularly.

## Conclusion

Ectopic adrenocortical adenomas are a rare condition, with only 31 cases reported to date. The majority of these cases were identified through the detection of ectopic masses via CT or MRI, followed by surgical resection and subsequent histopathological examination confirming the diagnosis of ectopic adrenocortical adenomas. Functional localization diagnostics were conducted in only a few cases. Due to the insidious nature of the symptoms in the early stages of the disease, which can easily be overlooked, a delay in detection may significantly impact the patient's reproductive function. Therefore, it is imperative to perform functional localization tests for early diagnosis, and postoperative assessment of gonadal function is crucial to facilitate appropriate interventions to promote the restoration of gonadal function.

## Data Availability

All data generated or analysed during this study are included in this published article.

## References

[1] Raff H, Carroll T (2015). Cushing’s syndrome: from physiological principles to diagnosis and clinical care. J Physiol.

[2] Stewart PM, Newell-Price JDC, Melmed S, Polonsky KS, Larsen PR, Kronenberg HM (2016). The Adrenal Cortex. Williams textbook of endocrinology.

[3] Newell-Price J, Bertagna X, Grossman AB, Nieman LK (2006). Cushing’s syndrome. Lancet.

[4] Souverijns G, Peene P, Keuleers H, Vanbockrijck M (2000). Ectopic localisation of adrenal cortex. Eur Radiol.

[5] Liu Y, Jiang YF, Wang YL, Cao HY, Wang L, Xu HT, Li QC, Qiu XS, Wang EH (2016). Ectopic adrenocortical adenoma in the renal hilum: a case report and literature review. Diagn Pathol.

[6] Tajima T, Funakoshi A, Ikeda Y, Hachitanda Y, Yamaguchi M, Yokota M, Yabuuchi H, Satoh T, Koga M (2001). Nonfunctioning adrenal rest tumor of the liver: radiologic appearance. J Comput Assist Tomogr.

[7] Ayala AR, Basaria S, Udelsman R, Westra WH, Wand GS (2000). Corticotropin-independent Cushing’s syndrome caused by an ectopic adrenal adenoma. J Clin Endocrinol Metab.

[8] Wang XL, Dou JT, Gao JP, Zhong WW, Jin D, Hui L, Lu JM, Mu YM (2012). Laparoscope resection of ectopic corticosteroid-secreting adrenal adenoma. Neuro Endocrinol Lett.

[9] Tong A, Jia A, Yan S, Zhang Y, Xie Y, Liu G (2014). Ectopic cortisol-producing adrenocortical adenoma in the renal hilum: histopathological features and steroidogenic enzyme profile. Int J Clin Exp Pathol.

[10] Hao Z, Ding J, Huo L, Luo Y (2022). ACTH-independent Cushing’s syndrome caused by an ectopic adrenocortical adenoma in the renal hilum. Diagnostics (Basel).

[11] Ilias I, Sahdev A, Reznek RH, Grossman AB, Pacak K (2007). The optimal imaging of adrenal tumors: a comparison of different methods. Endocr Relat Cancer.

[12] Israel GM, Korobkin M, Wang C, Hecht EN, Krinsky GA (2004). Comparison of unenhanced CT and chemical shift MRI in evaluating lipid-rich adrenal adenomas. AJR Am J Roentgenol.

[13] Chen S, Li R, Zhang X, Lu L, Li J, Pan H, Zhu H (2018). Combined ovarian and adrenal venous sampling in the localization of adrenocorticotropic hormone-independent ectopic Cushing syndrome. J Clin Endocrinol Metab.

[14] Carey RM, Mantero F, Murad MH, Reincke M, Shibata H, Stowasser M, Young WF, Funder JW (2016). The management of primary aldosteronism: case detection, diagnosis, and treatment: an endocrine society clinical practice guideline. J Clin Endocrinol Metab.

[15] Young WF (2007). Primary aldosteronism: renaissance of a syndrome. Clin Endocrinol (Oxf).

[16] Young WF, Stanson AW (2009). What are the keys to successful adrenal venous sampling (AVS) in patients with primary aldosteronism?. Clin Endocrinol (Oxf).

[17] Sanli Y, Garg I, Kandathil A, Kendi T, Zanetti MJB, Kuyumcu S, Subramaniam RM (2018). Neuroendocrine Tumor Diagnosis and Management: 68Ga-DOTATATE PET/CT. AJR Am J Roentgenol.

[18] Yami Channaiah C, Karlekar M, Sarathi V, Lila AR, Ravindra S, Badhe PV, Malhotra G, Memon SS, Patil VA, Pramesh CS, Bandgar T (2023). Paediatric and adolescent ectopic Cushing’s syndrome: systematic review. Eur J Endocrinol.

[19] Mete O, Asa SL, Giordano TJ, Papotti M, Sasano H, Volante M (2018). Immunohistochemical biomarkers of adrenal cortical neoplasms. Endocr Pathol.

[20] Rindi G, Mete O, Uccella S, Basturk O, La Rosa S, Brosens LAA, Ezzat S, de Herder WW, Klimstra DS, Papotti M, Asa SL (2022). Overview of the 2022 WHO classification of neuroendocrine neoplasms. Endocr Pathol.

[21] Ding J, Tong A, Hacker M, Feng M, Huo L, Li X (2022). Usefulness of 68 Ga-Pentixafor PET/CT on Diagnosis and management of Cushing syndrome. Clin Nucl Med.

[22] Concepción-Zavaleta MJ, Armas CD, Quiroz-Aldave JE, García-Villasante EJ, Gariza-Solano AC, Durand-Vásquez MDC, Concepción-Urteaga LA, Zavaleta-Gutiérrez FE (2023). Cushing disease in pediatrics: an update. Ann Pediatr Endocrinol Metab.

[23] Fourman LT, Fazeli PK (2015). Neuroendocrine causes of amenorrhea–an update. J Clin Endocrinol Metab.

[24] Zhang X, Wei Y, Li X, Li C, Zhang L, Liu Z, Cao Y, Li W, Zhang X, Zhang J, Shen M, Liu H (2022). The corticosterone-glucocorticoid receptor-AP1/CREB axis inhibits the luteinizing hormone receptor expression in mouse granulosa cells. Int J Mol Sci.

[25] Tabarin A, Assié G, Barat P, Bonnet F, Bonneville JF, Borson-Chazot F, Bouligand J, Boulin A, Brue T, Caron P, Castinetti F, Chabre O, Chanson P, Corcuff JB, Cortet C, Coutant R, Dohan A, Drui D, Espiard S, Gaye D, Grunenwald S, Guignat L, Hindie E, Illouz F, Kamenicky P, Lefebvre H, Linglart A, Martinerie L, North MO, Raffin-Samson ML, Raingeard I, Raverot G, Raverot V, Reznik Y, Taieb D, Vezzosi D, Young J, Bertherat J (2022). Consensus statement by the French Society of Endocrinology (SFE) and French Society of Pediatric Endocrinology & Diabetology (SFEDP) on diagnosis of Cushing’s syndrome. Ann Endocrinol (Paris).

[26] Magiakou MA, Mastorakos G, Oldfield EH, Gomez MT, Doppman JL, Cutler GB, Nieman LK, Chrousos GP (1994). Cushing’s syndrome in children and adolescents. Presentation, diagnosis, and therapy. N Engl J Med.

[27] Rabin DS, Johnson EO, Brandon DD, Liapi C, Chrousos GP (1990). Glucocorticoids inhibit estradiol-mediated uterine growth: possible role of the uterine estradiol receptor. Biol Reprod.

[28] Gordon CM, Ackerman KE, Berga SL, Kaplan JR, Mastorakos G, Misra M, Murad MH, Santoro NF, Warren MP (2017). Functional hypothalamic amenorrhea: an endocrine society clinical practice guideline. J Clin Endocrinol Metab.

[29] Endo M, Fujii H, Fujita A, Takayama T, Matsubara D, Kikuchi T, Manaka S, Mori H (2021). Ectopic adrenocortical adenoma in the renal hilum mimicking a renal cell carcinoma. Radiol Case Rep.

[30] Zhang J, Liu B, Song N, Lv Q, Wang Z, Gu M (2016). An ectopic adreocortical adenoma of the renal sinus: a case report and literature review. BMC Urol.

[31] Ashikari D, Tawara S, Sato K, Mochida J, Masuda S, Mukai K, Turcu A, Nishimoto K, Yamaguchi K, Takahashi S (2019). Ectopic adrenal adenoma causing gross hematuria: steroidogenic enzyme profiling and literature review. IJU Case Rep.

[32] Baek J, Kim SH, Cho SH, Kim WH, Kim HJ, Ryeom HK, Yoon G (2022). Ectopic adrenal adenoma in renal sinus: a case report. J Korean Soc Radiol.

[33] Ren PT, Fu H, He XW (2013). Ectopic adrenal cortical adenoma in the gastric wall: case report. World J Gastroenterol.

[34] Kepes JJ, O'Boynick P, Jones S, Baum D, McMillan J, Adams ME (1990). Adrenal cortical adenoma in the spinal canal of an 8-year-old girl. Am J Surg Pathol.

[35] Mitchell A, Scheithauer BW, Sasano H, Hubbard EW, Ebersold MJ (1993). Symptomatic intradural adrenal adenoma of the spinal nerve root: report of two cases. Neurosurgery.

[36] Cassarino DS, Santi M, Arruda A, Patrocinio R, Tsokos M, Ghatak N, Quezado M (2004). Spinal adrenal cortical adenoma with oncocytic features: report of the first intramedullary case and review of the literature. Int J Surg Pathol.

[37] Karikari IO, Uschold TD, Selznick LA, Carter JH, Cummings TJ, Friedman AH (2006). Primary spinal intramedullary adrenal cortical adenoma associated with spinal dysraphism: case report. Neurosurgery.

[38] Schittenhelm J, Ebner FH, Harter P, Bornemann A (2009). Symptomatic intraspinal oncocytic adrenocortical adenoma. Endocr Pathol.

[39] Rodriguez FJ, Scheithauer BW, Erickson LA, Jenkins RB, Giannini C (2009). Ectopic low-grade adrenocortical carcinoma in the spinal region: immunohistochemical and molecular cytogenetic study of a pediatric case. Am J Surg Pathol.

[40] Makino K, Kojima R, Nakamura H, Morioka M, Iyama K, Shigematsu K, Kuratsu J (2010). Ectopic adrenal cortical adenoma in the spinal region: case report and review of the literature. Brain Tumor Pathol.

[41] Skórka A, Moszczyńska E, Kot K, Roszkowski M, Jurkiewicz E, Grajkowska W, Pronicki M, Pilecki O, Szalecki M (2015). Ectopic virilising adrenocortical tumour in the spinal region in an 8 year-old boy: a case report and review of the literature. Ital J Pediatr.

[42] Konstantinov AS, Shelekhova KV (2016). Ektopicheskaya adrenokortikal'naya adenoma pozvonochnogo kanala: nablyudenie iz praktiki i obzor literatury [Ectopic adrenal cortical adenoma in the spinal canal: A case report and a review of the literature]. Arkh Patol.

[43] Woo HS, Lee KH, Park SY, Han HS, Yoon CJ, Kim YH (2007). Adrenal cortical adenoma in adrenohepatic fusion tissue: a mimic of malignant hepatic tumor at CT. AJR Am J Roentgenol.

[44] Shin YM (2010). Hepatic adrenal rest tumor mimicking hepatocellular carcinoma. Korean J Hepatol.

[45] Yoon JH, Kim SH, Kim MA, Han JK, Choi BI (2010). MDCT and Gd-EOB-DTPA enhanced MRI findings of adrenal adenoma arising from an ectopic adrenal gland within the liver: radiologic-pathologic correlation. Korean J Radiol.

[46] Park WY, Seo HI, Choi KU, Kim A, Kim YK, Lee SJ, Lee CH, Huh GY, Park DY (2017). Three cases of adrenocortical tumors mistaken for hepatocellular carcinomas/diagnostic pitfalls and differential diagnosis. Ann Diagn Pathol.

[47] Cho YS, Kim JW, Seon HJ, Cho JY, Park JH, Kim HJ, Choi YD, Hur YH (2019). Intrahepatic adrenocortical adenoma arising from adrenohepatic fusion mimicking hepatic malignancy: two case reports. Medicine (Baltimore).

[48] Chen J, Wan X, Lu Y, Wang W, Zhao D, Lu Z, Mao Y, Chen J (2021). An ectopic adrenocortical oncocytic adenoma in the liver highly mimicking hepatocellular carcinoma: case report and literature review. Diagn Pathol.

